# Design of a Knowledge Evaluation Questionnaire for Dental Specialists on Preservation and Extraction Indications of the First Permanent Molars

**DOI:** 10.30476/DENTJODS.2021.87989.1305

**Published:** 2022-03

**Authors:** Farnaz Farrokhi, Hamidreza Pakshir, Maryam Karandish, Mehrdad Askarin

**Affiliations:** 1 Student Research Committee, School of Dentistry, Shiraz University of Medical Sciences, Shiraz, Iran; 2 Orthodontic Research Center, Dept. of Orthodontics, School of Dentistry, Shiraz University of Medical Sciences, Shiraz, Iran; 3 Dept. of Orthodontics, School of Dentistry, Shiraz University of Medical Sciences, Shiraz, Iran; 4 Dept. of Community Medicine, School of Medicine, Medicinal and Natural Products Chemistry Research Center, Shiraz University of Medical Sciences, Shiraz, Iran

**Keywords:** Questionnaire and Survey, First Permanent Molar, Tooth Extraction, Knowledge

## Abstract

**Statement of the Problem:**

The first permanent molar (FPM) teeth are the most important elements of mastication and are crucial in the improvement of functionally proper occlusion. However, in childhood, these teeth are most susceptible to caries. The loss of an FPM in a child can cause changes in the dental arches. These changes can occur throughout a person’s life. In such cases, the dentists and dental specialists need to decide whether to preserve or extract the FPM.

**Purpose:**

This study aimed to evaluate the extent of knowledge of dental specialists in Shiraz (Iran) on clinical guidelines for the preservation and extraction indications of FPMs.

**Materials and Method:**

The authors developed a dedicated questionnaire for the purpose of knowledge evaluation. A total of 6 orthodontists and 15 dental specialists, respectively confirmed the validity and reliability of the questionnaire. The 19-item questionnaire covered topics such as demographic data, preservation criteria for FPM teeth, and indications for FPM extraction. The survey was carried out across six dental disciplines in Shiraz (Iran) during July-August 2018. The data were analyzed using the SPSS software (version 22.0) with the dependent sample *t* test and one-way ANOVA. *p* Value< 0.05 was considered statistically significant.

**Results:**

Out of 89 dental specialists, 64 participants (53% male, 47% female) completed the questionnaire. The mean knowledge score for all participants was 10.09±3.93 (maximum of 19). The level of knowledge had a significant and inverse correlation with age (*p*< 0.001) and years of experience (*p*= 0.017). It also had a significant relationship with dental specialization (*p*< 0.001).

**Conclusion:**

The overall level of knowledge of the specialists was insufficient, except for the pedodontists and orthodontists. A re-education training program for dental specialists is strongly recommended.

## Introduction

From the developmental and functional viewpoint, the first permanent molars (FPMs) are undoubtedly the most important teeth in a normal and balanced occlusion [ 1 ]. The importance of FPMs is regarded to their key role in preserving dento-facial harmony and masticatory function [ 2 ]. FPMs are the first permanent teeth to erupt in the oral cavity. However, due to their location in the dental arch, it is difficult to keep them clean. The long calcification period from birth to infancy and parents’ unawareness of the time of eruption of the FPMs and of their importance in the dentition, contribute to the fact that FPMs are susceptible to dental caries [ 3 ]. According to previous studies, both the upper and lower FPMs are highly vulnerable teeth to dental caries and hypoplasia [ 4 - 5 ]. Dental caries can be prevented if appropriate fluoride therapy (in the form of toothpaste, varnish, and fissure sealant) based on the needs of the patients is applied [ 6 ]. Determination of a suitable treatment method for badly decayed and hypo-plastic FPMs requires consideration of various factors. For example, the severity of a toothache, the degree of pulp maturation, the extent of crown destruction, the status of the developing dentition, child’s parents’ attitude toward oral dental care, and the ability of the patient to tolerate a lengthy treatment under local anesthesia. Hence, some clinicians defend the early extraction of these teeth while others prefer to restore even an extensively decayed FPM [ 7 ]. However, under certain clinical conditions, the extraction of extensively carious FPMs should be considered. Such conditions are hypoplastic FPMs, heavily restored FPMs where premolars are perfectly healthy, apical pathosis or endodontically treated FPMs, crowding at the distal aspect of the arches and third molars with reasonable form in a reasonable position, skeletally divergent malocclusions (dolichofacial vertical pattern), and anterior open bite malocclusion [ 8 ]. 

The timing and overall approach to FPMs extraction should be tailored to different occlusal relationships [ 8 ]. Nevertheless, the ideal stage for the extraction of the FPM is at the age of 8-10 years [ 9 ]. According to the latest guideline, the best time to extract FPM is after the eruption of lateral incisor, but before the eruption of the second permanent molar and/or the second premolar [ 10 ]. When an FPM with poor prognosis is extracted during this period, it has been claimed to cause mesial movement of the permanent second molar into the FPM region and thus creating the most ideal contact relationship with the permanent second premolars [ 11 ]. On the other hand, studies have also shown that early loss of FPM might accelerate the development of the third molar on the extracted side compared to that of the contralateral teeth [ 12 ]. FPMs extraction might also create more space for the eruption of the third molar and its movement into a better position [ 13 ]. Additionally, cases involving the extraction of FPMs often result in comprehensive orthodontic treatment; hence, determining the appropriate extraction timing can considerably facilitate and simplify the subsequent fixed orthodontic therapy [ 14 ]. However, FPMs extraction at an older age will lead to undesirable and insufficient space closure resulting in orthodontic malocclusion [ 11 ], in turn leading to contrary effects on the dental arch in both occlusion and function. These include reduced local function, tipping of adjacent teeth toward the extraction site, midline deviation, supra-eruption of opposing teeth, and unilateral chewing habit [ 3 ]. 

In their guideline, Cobourne *et al*. showed that enforced extraction of FPMs in children might be required. Therefore, it is important to determine the existence of any underlying malocclusion well before extraction. This guideline suggests that a compensating extraction (the extraction of the opposing tooth in the other arch) of an upper FPM is indicated after extraction of the lower FPM. However, routine compensating extraction of a lower FPM after the enforced extraction of the upper FPM is not recommended [ 10 ]. The balancing extraction (the extraction of the contralateral tooth in the same arch) of a sound FPM has been suggested to preserve the arch symmetry [ 10 ]. Currently, the balancing extraction of a sound FPM with the sole purpose of preserving the dental centerline is hardly justifiable [ 10 ]. Class-III malocclusions are often hard to manage; hence, balancing and compensating extractions are not indicated [ 10 ].

The present study aimed to evaluate the extent of knowledge of dental specialists in Shiraz (Iran) on clinical guidelines for the preservation and extraction indications of FPMs. 

## Materials and Method

The present cross-sectional study was carried out during July-August 2018 in Shiraz, Iran. Due to the unavailability of an appropriate data collection tool, a dedicated questionnaire was developed by the authors to evaluate the level of knowledge of dental specialists on clinical guidelines for the preservation and extraction indications of FPM. The content of the questionnaire was defined based on a review of various articles. 

A list of dental specialists was obtained from the registry of Shiraz University of Medical Sciences (SUMS) from which 113 specialists practicing in Shiraz were identified. Subsequently, the survey was carried out among six dental disciplines, namely endodontics, pedodontics, prosthodontics, orthodontics, oral and maxillofacial surgery and restorative dentistry. The inclusion criteria were specialization in one of the above-mentioned dental disciplines and place of practice in Shiraz. The exclusion criteria were general dental practitioners, incomplete questionnaire, and unwillingness to participate. Accordingly, from 113 identified specialists, 89 were recruited into the study. 

### The questionnaire

The questionnaire consisted of 19 items divided into three categories, namely demographic data, preservation criteria for FPM teeth, and indications for FPM extraction. Demographic characteristics included variables such as sex, type of university, university of graduation, graduation date, years of experience, specialty and place of practice. The category on the preservation of FPM teeth included 8 items that covered topics such as the role of FPMs in cheek esthetics, the reasons for not extracting FPMs before the age of 8 years and the cons-equences of extracting FPMs at or after the final stage of second permanent molar eruption. The category on FPM extraction criteria included 11 items covering topics such as the required actions before extracting FPM, the best time for FPMs extraction and balancing and compensating extractions (Table 1). The knowledge score was determined by counting the total number of correct answers given by the participants. The scores ranged from 0 to 19; a higher score indicated better knowledge.

**Table 1:** The knowledge questionnaire and the expected answer to each question

No.	Question	Agree	Do not know	Disagree
1	If you face a child presenting with a developing dentition affected by one or more first permanent molars of poor prognosis:			
1-1	First permanent molars can be extracted and substitute with second permanent molar teeth with a proper treatment plan.	*		
1-2	Before tooth extraction, radiographic screen should be done to become sure about other molars position and their natural formation.	*		
2	If there are favorable conditions, balancing and compensating extraction of first permanent molars will be carried out to preserve the occlusal relationship and symmetric dental arch.	*		
3	Which of the following statements have an impact on deciding for the balancing or compensating extraction of the first permanent molars?			
3-1	The overall and the long-term prognosis of the first permanent molar	*		
3-2	The existence of second and third permanent molars	*		
3-3	The type of the present malocclusion	*		
4	If the enforced extraction of a lower first permanent molar is required, the compensating extraction of an upper first permanent molar should be recommended. It prevents over eruption of upper first permanent molar	*		
5	The compensating extraction of a lower first permanent molar has not been recommended when extraction of the upper first permanent molar is required.	*		
6	Balancing extraction of a sound first permanent molar has been recommended to prevent midline deviation.			*
7	The timing for lower first permanent molar extraction is more important than the upper first permanent molar extraction timing. Because the migration of the lower second permanent molar is unpredictable.	*		
8	The most favorable chronological age for enforced extraction of lower first permanent molar is 8-10 years, after the eruption of the lateral incisors but before the eruption of the second permanent molar and/or second premolar.	*		
9	First permanent molar extraction before 8year is not suggested because of:			
9-1	Absence of radiographic evidence of third permanent molar	*		
9-2	Second premolar migration to the space of extracted tooth	*		
9-3	Lingual drifting of anterior teeth and increased overbite	*		
10	Extraction of first permanent molars at the final stage of second permanent molar eruption or after it can cause:			
10-1	Rotation and mesial tipping of second permanent molar to the space of the extracted tooth	*		
10-2	Distal tipping of the second premolar to the space of the extracted tooth	*		
10-3	Undesirable teeth contacts and occlusal relationship	*		
11	In class III cases if the enforced extraction of lower first permanent molar become needed the balancing and compensating extraction will be carried out.			*
12	First permanent molars have a key role in cheeks esthetic. Cheeks appear full and vibrant in the presence of first permanent molars.	*		

### Validity and Reliability Tests

To confirm the validity of the knowledge evaluation questionnaire, the 19 items were initially reviewed by 6 orthodontists (academic staff) and based on their feed- back initial modifications were implemented to shorten or clarify the questions. They unanimously agreed that the questions were appropriate for the targeted group. Hence, the questionnaire was used without a question being modified or removed. To confirm the reliability of the knowledge evaluation questionnaire, the test-retest method was used. A total of 15 dental specialists (orthodontists (n=2), pedodontists (n=2), prosthodontists (n= 4), restorative dentists (n=3), endodontists (n=3), oral and maxillofacial surgeon (n=1)) were randomly selected to review the questions twice with an interval of 1 month. It is notable to say that these participants did not take part in the main study. The results from both tests were analyzed and the correlation coefficient was determined to confirm the reliability of the questionnaire.

### Data Analysis

The data were analyzed using the SPSS software, version 22.0. Descriptive statistics such as mean and standard deviation, absolute frequency and relative frequency were determined. The Spearman non-parametric correlation test was used to investigate the relationship between knowledge, age and years of experience. The one-way ANOVA and dependent sample t test were used to compare the knowledge score with respect to the field of dental specialty as well as demographic characteristics. Additionally, the post-hoc Tukey’s test was used to compare the extent of knowledge between the dental specialty groups. The data were presented as mean±SD and *p*< 0.05 was considered statistically significant.

## Results

From 89 recruited dental specialists, 64 (71.9%) participants fully completed the knowledge evaluation questionnaire. The dental specializations of the participants are shown in Table 2.

**Table 2:** The number and the percentage of the participants with respect to dental specialization

Specialty	Number (%)
Endodontists	12 (18.8)
Prosthodontists	13 (20.3)
Restorative dentists	11 (17.2)
Orthodontists	10 (15.6)
Pedodontists	10 (15.6)
Oral and maxillofacial surgeons	8 (12.5)
Total	64 (100)

The participants had a mean age of 37.5±6.4 years (range: 28-58 years), male 34 (53.1%), and work experience 7.06±4.19 years (range: 1-17 years). In terms of education, 38 (59.4%) participants were graduates from SUMS and 31 (48.44%) graduated during 2011-2016. Most of the participants worked in dental faculty clinics 26 (40.6%) and 40 (62.5%) were faculty staff at SUMS (Table 3). The mean score of the level of knowledge for all participants was 10.09±3.93 (range: 1-18). Item 1-2 had the highest (95.3%) and item 6 the lowest (3.1%) score for the correct answers. The Kolmogorov-Smirnov test was used to determine the normality of the demographic variables and the level of knowledge.

**Table 3:** Demographic characteristics of the participants

Demographic variables		Number
Sex	Male	34
	Female	30
University	SUMS	38
	Other national universities	26
Graduation year	1999-2004	7
	2005-2010	26
	2011-2016	31
Work experience	≤5	23
	5-10	30
	>10	10
Workplace	Private practice	21
	Private and governmental dental clinic	17
	Dental faculty clinic	26
Faculty staff	SUMS	40
	Islamic Azad University	4
	No faculty member	20

The correlation between knowledge, age, and years of experience was examined. The results showed that the level of knowledge had a significant inverse correlation with age (*p*< 0.001, correlation coefficient= -0.494) and the years of experience (*p*= 0.011, correlation coefficient= -0.317). Furthermore, all female participants and those graduated from SUMS had the highest level of knowledge (*p*=0.046, *p*= 0.029, respectively). The participants graduated during 2011-2016 and with <5 years of experience scored the highest level of knowledge (*p*= 0.005, *p*= 0.009, respectively) (Table 4).

**Table 4:** The relationship between knowledge level and demographic variables

Demographic variables		Number	Knowledge score (Mean±SD)	*p* Value
Sex	Male	34	9.17±4.18	0.046*
	Female	30	11.13±3.39	
University	SUMS	38	10.97±3.92	0.029*
	Other national universities	26	8.80±3.63	
Graduation Year	1999-2004	7	10.00±4.28	0.005*
	2005-2010	26	8.30±3.69	
	2011-2016	31	11.61±3.49	
Work experience	≤5	23	11.95±3.77	0.009*
	5-10	30	8.66±3.68	
	>10	10	10.30±3.65	
Workplace	Private practice	21	10.28±3.16	0.658
	Private and governmental dental clinic	17	9.35±7.57	
	Dental faculty clinic	26	10.46±4.62	
Faculty staff	SUMS	40	9.87±4.45	0.775
	Islamic Azad University	4	11.25±3.77	
	No faculty member	20	10.30±2.79	

* Statistical significance

The level of knowledge had a significant correlation with the type of dental specialization (*p*<0.001). Amongst all dental specialists, pedodontists and endodontists had the highest [ 18 ] and the lowest [ 11 ] knowledge score, respectively (Table 5). The post-hoc Tukey’s test was used to determine the difference in knowledge scores among the six dental groups (Table 6).

**Table 5:** The mean, maximum and minimum of knowledge scores within each dental specialty

Specialty	Number	Knowledge score*(Mean±SD)	Score	
			Maximum	Minimum
Endodontists	12	5.58±2.71	11	1
Prosthodontists	13	11.15±2.57	15	6
Restorative dentists	11	10±3.68	15	3
Orthodontists	10	11.5±1.71	15	9
Pedodontists	10	14±2.58	18	8
Oral and maxillofacial surgeons	8	8.62±4.5	15	1

* p= 0.000

**Table 6:** Group comparisons between each dental specialty

Dental specialist groups		Sig.
Endodontists	Prosthodontists	0.000*
	Restorative dentists	0.011*
	Orthodontists	0.000*
	Pedodontists	0.000*
	Oral and maxillofacial surgeons	0.247
Prosthodontists	Restorative dentists	0.935
	Orthodontists	1.000
	Pedodontists	0.232
	Oral and maxillofacial surgeons	0.430
Restorative dentists	Orthodontists	0.862
	Pedodontists	0.039*
	Oral and maxillofacial surgeons	0.921
Orthodontists	Pedodontists	0.438
	Oral and maxillofacial surgeons	0.347
Pedodontists	Oral and maxillofacial surgeons	0.005*

* Statistical significance

The endodontists scored the lowest and their score significantly differed from all other disciplines, except for those of the oral and maxillofacial surgeons.

The percentage of dental specialists, per discipline, who agreed with each item of the questionnaire, is shown in Table 7. More than 80% of the pedodontists agreed with the rationale of at least 10 items of the questionnaire while more than 80% of the endodontists only agreed with the rationale of 1 item.

**Table 7:** The percentage of dental specialists in each dental discipline who agreed with each item of the questionnaire

#		Questions	Endo	Prostho	Resto	Ortho	Pedo	OMF
1	In the case of a child with a developing dentition affected by one or more first permanent molars of poor prognosis:							
	1.1	The first permanent molars can be extracted and substituted by second permanent molar teeth in accordance with a proper treatment plan							
			50.0%	92.3%	81.8%	90.0%	100.0%	62.5%
	1.2	Before tooth extraction, radiographic screening is required to determine the other molars position and their stage of development.							
			83.3%	100.0%	100.0%	100.0%	90.0%	100.0%
2	If favorable conditions are present, balancing and compensating extraction of the first permanent molars can be carried out to preserve the occlusal relationship and symmetric dental arch.							
			16.7%	46.2%	18.2%	50.0%	70.0%	37.5%
3	Which of the following statements are relevant in deciding on balancing or compensating extraction of the first permanent molars?							
	3.1	The overall and long-term prognosis of the first permanent molar							
			66.7%	84.6%	81.8%	100.0%	100.0%	75.0%
	3.2	The presence of second and third permanent molars							
			50.0%	84.6%	81.8%	90.0%	100.0%	75.0%
	3.3	The type of underlying malocclusion							
			33.3%	84.6%	72.7%	100.0%	100.0%	75.0%
4	If the enforced extraction of a lower first permanent molar is required, the compensating extraction of an upper first permanent molar is recommended. It prevents over-eruption of the upper first permanent molar.							
			8.3%	15.4%	0.0%	30.0%	70.0%	12.5%
5	The compensating extraction of a lower first permanent molar is not recommended when extraction of the upper first permanent molar is indicated.							
			41.7%	38.5%	81.8%	40.0%	60.0%	50.0%
6	Balancing extraction of a sound first permanent molar is recommended to prevent midline deviation.							
			8.3%	0.0%	0.0%	0.0%	10.0%	0.0%
7	The timing for lower first permanent molar extraction is more important than it is for the upper first permanent molar because the migration of the lower second permanent molar is unpredictable.							
			8.3%	46.2%	36.4%	70.0%	80.0%	12.5%
8	The most favorable chronological age for enforced extraction of lower first permanent molar is 8-10 years, after the eruption of the lateral incisors but before the eruption of the second permanent molar and/or second premolar.							
			41.7%	53.8%	18.2%	90.0%	66.7%	25.0%
9	First permanent molar extraction before the age of 8 years is not indicated because of:							
	9.1	Absence of radiographic evidence of the presence of the third permanent molar							
			25.0%	61.5%	63.6%	60.0%	100.0%	50.0%
	9.2	Second premolar migration into the space of the extracted tooth							
			0.0%	53.8%	54.5%	40.0%	80.0%	25.0%
	9.3	Lingual drifting of anterior teeth and increased overbite							
			8.3%	76.9%	72.7%	70.0%	60.0%	37.5%
10	Extraction of first permanent molars at or after the final stage of second permanent molar eruption can cause:							
	10.1	Rotation and mesial tipping of second permanent molar toward the space of the extracted tooth							
			50.0%	92.3%	90.9%	100.0%	80.0%	62.5%
	10.2	Distal tipping of the second premolar toward the space of the extracted tooth							
			8.3%	61.5%	36.4%	40.0%	70.0%	37.5%
	10.3	Undesirable tooth contacts and occlusal relationships							
			41.7%	69.2%	63.6%	40.0%	80.0%	62.5%
11	If enforced extraction of the lower first permanent molar is needed in Class-III cases, balancing and compensating extractions are recommended.							
			0.0%	30.8%	0.0%	20.0%	50.0%	25.0%
12	The first permanent molars have a key role in cheek esthetics. Due to their presence, cheeks appear full and vibrant.							
			16.7%	23.1%	45.5%	20.0%	40.0%	37.5%

Endo: Endodontists, Prostho: Prosthodontists, Resto: Restorative dentists, Ortho: Orthodontists, Pedo: Pedodontists, OMF: Oral and maxillofacial surgeons

## Discussion

The present study aimed to evaluate the extent of kno-wledge of dental specialists on clinical guidelines about the preservation and extraction indications of FPM. Cobourne *et al*. [ 10 , 15 ] introduced “A Guideline for the Extraction of Fist Permanent Molars in Children” in 2009 and its updated version was published in 2014. To date, no studies have been conducted to evaluate the level of knowledge of dental specialists on this guideline. Therefore, a dedicated questionnaire was developed by the authors and its validity and reliability were confirmed. The results of the test-retest indicated a high correlation coefficient of 0.8; confirming the applicability of the questionnaire toward our objective. The scale for the mean score of the level of knowledge was categorized in five ranges, namely excellent (≥80%), very good (70% -79%), good (60%-69%), mediocre (50%-59%) and weak (<50%). The scores of our participants varied from weak to very good, depending on their field of specialty. Almost all the participants agreed on performing radiography when faced with a child, during the developed dentition stage, with one or more carious molar teeth with poor prognosis. However, they all scored the lowest on the item regarding the prerequisites for the balancing extraction of a sound FPM. The results showed a significant relationship between the level of knowledge and the field of dental specialization. The knowledge scores of the endodontists differed from other groups, except for the oral and maxillofacial surgeons. In addition, there was a statistical difference between the pedodontists and the restorative dentists as well as the oral and maxillofacial surgeons.

The level of knowledge among the dental specialists was ranked in the following descending order: pedodontists, orthodontists, prosthodontists, restorative dentists, oral and maxillofacial surgeons, and endodontists. Surprisingly, the orthodontists scored lower than the pedodontists; however, both scored higher compared to the other dental specialists. Note that FPMs as the key to balanced occlusion have a great importance in the reading lists of orthodontists and pedodontists. They are well aware of the consequences of the extraction of poor prognosis FPMs and the possible of their substitution with second permanent molars. Endodontists gained the lowest score due to the concept of restoration and preservation of adult molars and this may influenced their opinion. They had insufficient knowledge about the migration of the second premolar to the space created by the extracted FPM due to early extraction. According to the guideline, in the mandibular arch, if the FPM is extracted before the age of 8 years, the second premolar can drift distally into the extraction space, rotate, and tip [ 16 - 17 ]. Moreover, the endodontists did not have enough knowledge about managing the enforced extraction of FPMs in Class-III cases. The guideline asserted that Class-III cases are usually difficult to manage and the advice from an orthodontist is required prior to any FPMs extraction. In general, extraction of the upper molars should be avoided if (at all) possible, while balancing and compensating extractions are not recommended in Class-III cases. A tendency toward a larger open space for the second permanent molar has been observed in the lower arch of Class-III cases following FPM extraction [ 18 ].

According to the guideline, in a child with a developing dentition affected by one or more FPM of poor prognosis, attention should be given to elective extraction (balancing, compensating extractions) or enforced extraction. At this stage, compensating or balancing extractions of sound FPM should be considered as part of the treatment plan. Before the elective extraction of any teeth, a radiographic screening should be done to check for the position, presence, and normal formation of the developing permanent dentition [ 10 ]. Fortunately, all endodontists, oral and maxillofacial surgeons, prosthodontists, restorative dentists, and orthodontists were aware of this requirement.

The knowledge score of the oral and maxillofacial surgeons ranged from weak to good, whereas prosthodontists scored from weak to very good. Nonetheless, none of them agreed with the rationale for the balancing extraction of sound FPM. It should be noted that the balancing extraction of sound FPMs has been suggested for keeping arch symmetry [ 8 , 19 ]. Retrospective cohort studies have reported that the unilateral extraction of an FPM can be related to the development of both dental and skeletal arch asymmetries [ 1 , 20 ]. Evidence from other studies showed that the dental midline of any of the dental arches was not likely to be affected [ 21 - 22 ]. Presently, it is difficult to defend the balancing extraction of a sound FPM solely for maintaining a dental midline. 

The knowledge of the restorative dentists ranged from weak to very good. Nonetheless, they genuinely disagreed with the rationale regarding the balancing extraction, management of enforced extraction of FPM in Class-III cases, and recommendation for the extraction of the upper FPM when an enforced extraction of the lower FPM is required. The guideline stated that, generally, the compensating extraction of a maxillary FPM is indicated when the extraction of the mandibular FPM is needed [ 23 ]. Based on various retrospective cohort studies, extraction is done to avoid the over-eruption of an unopposed maxillary FPM, which can cause potential occlusal interferences and prohibits the favorable mesial movement of the erupting mandibular second permanent molar. With the aim of providing reliable evidence for the enforced extraction of the mandibular FPM, a randomized controlled trial established that compensating extraction of the maxillary FPMs should be performed .

The knowledge score of the orthodontists ranged from mediocre to good. They all agreed that the type of malocclusions, as well as the overall and long-term prognosis of the FPMs, had an impact on the decision for balancing or compensating extraction of these teeth. Various factors affect the decision whether an FPM is suggested for either a balancing or compensating extraction: (i) which of the FPM(s) require enforced extraction, (ii) the overall situation and long-term prognosis of the remaining FPM(s), and (iii) the current and developmental condition of the dentition (including third molars) and the primary malocclusion. Our results showed that all the orthodontists had an excellent level of knowledge about the rotation and mesial tipping of the second permanent molar into the space of the extracted tooth due to FPMs extraction at or after the final stage of the second permanent molar eruption. However, none of them agreed with the rationale for the balancing extraction of a sound FPM.

The knowledge of the pedodontists ranged from good to excellent. All pedodontists agreed that FPMs could be extracted and substituted with the second permanent molar teeth with a proper treatment plan when faced with one or more FPMs of poor prognosis in the developing dentition of a child. They all agreed that the overall and long-term prognosis of the FPM, the presence of second and third permanent molars, and the type of malocclusions had an impact on the decision for balancing or compensating extraction of the FPMs. They also agreed that FPM extraction before the age of 8 years should not be recommended in the absence of radiographic evidence of the third permanent molar. 

The results of the present study indicated that knowledge had a significant inverse correlation with age and years of working experience. Note that the majority of the participants were young and/or had less than 10 years of working experience; therefore, they were more interested in this topic than the senior specialists were. Consequently, they obtained a higher score for the level of knowledge. The low score of senior specialists may be due to the lack of such guidelines while they were at dental school. On the other hand, their engagement with continued professional development such as courses and updated could have been insufficient. Female participants had a higher level of knowledge than their male counterparts did. Dental specialists graduated from SUMS performed better than those graduated from other Iranian universities did. Note that the sample size of those from other Iranian universities was low. Hence, the comparison was only made based on two groups of graduates. Consequently, the result cannot be interpreted as to conclude the educational protocol of SUMS is better than other Iranian universities. Moreover, dental specialists graduated during 2011-2016 (i.e. <5 years of experience) showed a higher level of knowledge. 

The main strength of the present study is the fact that it is the first implementation and evaluation of the guideline. Hence, it can be considered as a benchmark for future studies. Since the study was conducted during the summer period, its main limitation was the unavailability of some specialists. As a direct result, the sample size in the present study was low. A nationwide study on the level of knowledge of the Iranian dentists is recommended to identify the shortcomings of the guideline. With some minor alterations, the designed questionnaire can also be used to evaluate the level of knowledge of general dentists. It is also recommended to design workshops on the importance of the guideline in order to enhance the level of knowledge of the specialists, particularly the endodontists, and the oral and maxillofacial surgeons. 

## Conclusion

The results of the present study showed that endodontists, prosthodontists, oral and maxillofacial surgeons, and restorative dentists had an insufficient level of knowledge on extraction indications of FPM with poor prognosis as well as its management in different types of malocclusions. Only the pedodontists and orthodontists demonstrated an adequate level of knowledge. Considering the above, it is anticipated that general dentists would score even lower. Therefore, in support of fresh dental graduates, it is recommended that the dental curriculum include indications for FPM extraction. Moreover, a re-education training program for dental specialists is strongly recommended.

## Conflict of Interest

There is no competing interest to declare. 
